# Transitional Pain Care in Quebec: Did We Forget Our Youths? A Brief Research Report

**DOI:** 10.3389/fpain.2022.885570

**Published:** 2022-05-27

**Authors:** Irina Kudrina, Gillian Bartlett, M. Gabrielle Pagé, Yoram Shir, Leon Tourian, Manon Choinière, Isabelle Vedel

**Affiliations:** ^1^Department of Family Medicine, McGill University, Montreal, QC, Canada; ^2^Department of Anesthesia, McGill University Health Center, Montreal, QC, Canada; ^3^Family and Community Medicine, School of Medicine, University of Missouri, Columbia, MO, United States; ^4^Department of Anesthesiology and Pain Medicine, Université de Montréal, Montreal, QC, Canada; ^5^Research Center of the Centre Hospitalier de l'Université de Montréal, Montreal, QC, Canada; ^6^Department of Psychiatry, McGill University Health Center, Montreal, QC, Canada

**Keywords:** adolescents and young adults, chronic pain, psychosocial factors, transitional care, model (5)

## Abstract

Adolescents and young adults (AYAs) represent a unique population with distinct psycho-social risks and care needs. About 10% of AYAs live with chronic pain (CP) and transition to adult pain care between 16 and 25 years of age. These transitions in care happen simultaneously with other bio-psycho-social changes and require flexible multi-disciplinary support models. As it stands, transitional pain care appears suboptimal, fragmented, and opportunistic in Quebec (Canada). The objective of this Brief Report is, therefore, to present our study findings and propose a multi-disciplinary transitional framework vision applicable to AYAs living with CP. Data were collected using a sequential-consensual qualitative design with a longitudinal participatory component. The consecutive stages of this work included an exploratory stage, semi-structured interviews with primary care providers, and inter-disciplinary deliberative stakeholder consultation groups. The deductive inductive thematic approach and the three-level Health Care Transition Research Consortium's theoretical framework were used to analyze the data. A representative group of stakeholders discussed findings from the first two steps, made fifteen actionable recommendations and formulated their vision of a transitional pain care model that can be further adapted in other settings. The study results present important insights into various psycho-social factors associated with transitional pain care for AYAs.

## Introduction

An estimated 9–12% of Canadians aged 12 to 44 years live with chronic pain (CP) condition (1). CP persists beyond 3 months (2) and disproportionately affects already vulnerable populations (3) resulting in a significant societal cost (4–6). A comprehensive conceptualization of CP involves an interplay of bio-psycho-socio-cultural factors and cognitive experiences resulting in sensory, affective, motivational responses, and expressions of suffering (2, 7). Adolescents and young adults (AYAs) living with CP represent a unique population with specific psycho-social risks and care needs (8, 9). It can even be argued that due to several modifiable risk factors affecting decades of their functional lives ahead, this population needs the utmost attention from the medical and research communities. Yet, for the public, CP is commonly associated with a visible injury or an advanced age thus limiting the development of pain-focused policies and initiatives targeting youths.

CP is known to influence the neuro- and social development of children and youths (10, 11), who might be survivors of traumatic experiences related to pediatric procedures (12), stressful life events (13), stigma related to pain, and mental health (14) that perpetuate CP into the adulthood (15). There is limited research on cultural and racial differences to understand AYAs' pain experiences and choices of pain management modalities (7). In a multi-cultural country like Canada, such a lack of visibility risks worsening already existing inequity and inequality in the allocation of resources for AYAs.

The last stage of transition and integration to adult services is almost a decade long (16–25 years of age) and happens simultaneously with transitions in vocational training and employment, social and family responsibilities, rendering AYAs prone to multiple stressors and poor clinical outcomes (16, 17), extensive use of emergency services (18), and overall worsening of psycho-social indicators (19). AYAs are known to be at a risk of new psychiatric diagnoses (20–22), experimentation with substances and high-risk behaviors (23–25), sexually transmitted infections (26, 27), sedentary lifestyles (28), and poor eating habits (29). In the pandemic context, loneliness and low education among youths are associated with increased use or initiation of cannabis, e-cigarettes, and binge drinking (30) and opioid and stimulant-associated mortality (31, 32). AYAs living with CP are at especially high risk of suicidality (33). Thus, this population presents with a distinct baseline risk profile that substantiates the need for an urgent shift in clinical thinking, interventions, and research focus.

The Canadian Association of Pediatric Health Centers (CAPHC) guideline (34) recommends that all young patients with chronic conditions should be registered with a community-based primary care provider (PCP). However, there is a very limited literature on post-transfer practices and almost no data on pain-relevant transitional multidisciplinary models of care (35) to involve PCPs longitudinally. In a review of the published scientific and gray literature (35), we identified that only three out of fifteen transitional models described at least some involvement of primary care. All identified programs were initiated by pediatric specialties with an overall limited adult practitioners' and PCPs' input.

Pediatric care ends when adolescents are discharged at the age of 18, which makes the small number of existing transitional programs unipolar and narrowly focused on the preparation for the transfer to adult services. As our understanding of the PCPs' role in transitions, including training requirements, is developing (36), the scope of the necessary knowledge and skills in adolescent and pain medicine remains unidentified. Confounding is the limited availability of PCPs in Canada and especially in Quebec where this study took place. Due to the lack of standardized transitional outcomes, identified programs were not systematically funded or evaluated. In addition, there is a paucity of evidence on dedicated CP transition pathways and the role of recipient adult services. However, there is an agreement in the literature that the transitional care provision should be developmentally appropriate (37) rather than age-based (38) and would account for a variety of psycho-social factors along the transition trajectory.

The objective of this Brief Report is, therefore, to present our study findings of the psycho-social factors relevant to the transitional pain care for AYAs and propose a multi-disciplinary transitional framework vision pertinent to the pain field and applicable to this important population segment.

## Methods

### Design

Data were collected using a sequential-consensual qualitative design (39) with a longitudinal participatory component (40, 41). The three consecutive stages included: *stage 1*, an exploratory stage that informed further steps, followed by *stage 2*, semi-structured interviews with twelve PCPs, and concluded with *stage 3*, three inter-disciplinary deliberative stakeholder consultations groups. Three AYAs patient-partners were involved longitudinally in the entirety of the project, directing its development, validating study findings, and helping with the understanding of the findings' significance.

### Framework

For data collection and further analysis, we applied hierarchical frameworks based on the CAPHC guideline (34) and Health Care Transition Research Consortium (HCTRC) theoretical approach (42). The CAPHC guideline is concerned with transitional processes, tools, and resources at the personal, clinical, and system levels. The HCTRC theoretical framework offers a comprehensive description of variables important for patients with complex care needs in the individual, family and social support, environmental, and healthcare system domains. We adopted this approach by categorizing our findings hierarchically and conceptualizing all results into the three domains: *individual, service*, and healthcare *system* levels. A detailed description of methodology and findings are provided elsewhere (35).

### Settings and Participants

The study involved a multi-disciplinary team of the McGill pain clinic [McGill University Healthcare Network (43)], primary care providers, allied professionals, and AYAs patient-partners. The second and third stages were informed by the preliminary findings from the first (exploratory) stage.

#### Sampling Strategy

In stage 1, a core clinical research team interviewed active AYA patients and their supporters during their clinical encounters for over 1 year.

In stage 2, the input from PCPs was sought. A random purposeful sampling strategy was employed (44, 45). Sample adequacy (46) was assured. We searched our administrative hospital database and sent invitations to more than 30 PCPs. The final sample resulted in 12 participants, aged 29–70, with a wide range of experiences (family medicine groups, emergency medicine, solo practice, private office) and a number of years in practice (3–40 years). Seven participants were men, and a majority were English-speaking (N-9).

In stage 3, three deliberative stakeholder consultation groups (47, 48) concluded the project: (i) clinicians, (ii) allied healthcare professionals (AHP), and (iii) AYAs and their supporters. We chose a purposive homogenous sampling technique (44, 45) to recruit individuals who possess specific characteristics: those who have clinical, non-clinical, and patient experiences in McGill Healthcare Network and are familial with different institutional policies and processes in place. The final group composition thus represented primary and tertiary care professionals at different stages of their careers (nurses, medical residents, staff clinicians, and a clinical director), seven AHP (clinical psychologists, social worker, public health researcher, physiotherapists, and an administrative director), and six AYAs and their supporters. As some qualitative studies might be concerned with a degree of data generalization, determining an appropriate sample size was important. Onwuegbuzie et al. (44) reviewed qualitative literature and showed that in most cases, a group size of 6–12 participants allows for sufficient opportunities to share rich insights, sustain discussions, and present a sufficiently large spectrum of opinions. In homogenous sampling, to realistically reach a data saturation point, a total of 12–20 data sources might be necessary. Thus, to achieve informational redundancy and data saturation in our three groups, we selected a sample size of 6–7 participants per group.

### Data Collection

In stage 1, data collection was informed by the CAPHC (34) guideline. The team evaluated AYA patients' feedback during their clinical encounters and analyzed existing transitional literature, tools, and protocols. All findings were discussed, summarized, and conceptualized during the meetings with a larger clinical research team and patient partners. Two bilingual (English and French) interview guides (49) were constructed based on the emerged themes, one for stage 2 and one for stage 3.

Each participant chose an alias and signed informed consent. Individual interviews and group deliberations were recorded; transcripts were anonymized and transcribed verbatim.

In stage 2, semi-structured interviews explored how PCPs structured their practices, their comfort level providing transitional and pain care for AYAs with CP, and what role they can play in transitions.

In stage 3, to transfer knowledge (39) from stages 1 and 2, participants received a summary of the study findings and then were asked to deliberate on transitional guidelines and endorse relevant recommendations. We thus included decision-makers and key stakeholders in the shared decision-making process (48, 50), mitigating power differentials.

### Data Analysis

We performed an iterative deductive inductive thematic analysis of the transcribed bilingual data collected in stages 2 and 3 using QRS International's NVivo11 software (2015, London) (51). The three-level (*individual, service*, and *healthcare system (system)*) transitional framework was adopted. The deductively coded data were categorized into the corresponding framework domains. The inductive analysis allowed for the emergence of the new codes. The reviewers iteratively identified and discussed emerging themes and drew conclusions.

## Results

Data from stages 1 and 2 were used to inform stage 3. We present a summary of the main findings from stages 2 and 3 supported by the quotes from participants. At the *individual* (patient) level, all participants agreed that the integration of adult services and building youths' capacity for self-management should be the focus. At the *service* level, the improvement in the inter-disciplinary care coordination and communications were noted in all three stages. Overall, our findings indicate that the most important barriers to overcome were identified at the *system* level. Even individual and service variables were frequently discussed as a function of the *system* level barrier and facilitators. Due to a high degree of fragmentation of the medical care provision, the need for more robust and structured transitional care was recognized by all participants. Unexpectedly, the initial concern about mental health and addiction, discussed in the exploratory and interview with PCPs stages, was not included in the final set of recommendations. Below, we summarize each level in more detail as matched to chosen CAPHC guideline recommendations and by applying HCTRC theoretical framework variables as appropriate for our setting.

### Individual (Patient) Level

#### CAPHC Recommendation-1

Individual transition planning was discussed by the clinicians and AHP groups. Their views converged stating that due to the pediatric and adult care models differences, AYAs' would benefit from a patient peer support network to undergo “*an orientation session [with] their parents where the whole process can be explained, and we're (pain service team) up front setting expectations about what this service is about…kind of concrete protocols or concrete steps that could be implemented at the system level to facilitate that transition”* (Earth, AHP). Transitional planning was strongly supported by the PCPs who felt that AYAs live through very complex times when “*…nothing is settled. It's shifting sands in every way, within their personal life, their professional life, their studies, their love life, their whatever, their family relations… So, … they're much more vulnerable to screwing up”* (Dr. Office). Another PCP emphasized how AYA's planning capacity could still be in development, “*I think it has to do with how their brains work, and they're more impulsive, and they're more in their present. So, if it's not in the front of their awareness, they, other things are more important, and that's what they focus on”* (Dr. Diego). These sentiments were echoed by the stage 3 group participants, who saw transition planning as multi-faceted and continued with the adult provider teams.

#### CAPHC Recommendation-2–3

Clinicians and patient groups agreed on the need for accommodations of the AYAs' vocational and working schedules, recommended a telephone helpline (clinicians), and implementation of “drop-in hours” similar to walk-in clinics (patients). Clinicians emphasized that “*… it's even more important sometimes in this group of people who do have chronic pain, who miss a lot of schools, who miss a lot of working opportunities, to give them the flexibility to have later evenings, or early mornings, that we promote that they don't miss school or don't miss work”* (Paris). AYAs insisted that the timely supporting documentation plays a crucial role in validating their limitations and obtaining disability-related accommodations “*so that I can keep up with the other students and sometimes just to get those kinds of letters and stuff can take months… if I have midterms, if I have finals in university, I need them right now… getting documents, simple documents and such becomes really, really tough.”* (July).

### Service Level

#### CAPHC Recommendation-5

One of the most contentious points was the need for improved communication strategies to ensure “*safe, caring and effective transitions*.” One of the most discussed solutions was a shared electronic online platform accessible to patients and healthcare providers that “*…would house information… [be] patient friendly… [with] a visual component that might be more interactive”* (Rio, clinician). Such technology could be multi-functional and serve for the confidential information exchange, as an administrative tool. There might be a patient portal offering virtual orientation sessions, the ability to contact the team, and assisting with a standardized transition process by providing “…*a web-based referral form that obtains the essential information needed by the pain treatment clinic*” (Titan, AHP). Such a platform was also seen as a safe space for case discussions and knowledge exchange with “*…the hub experts of the pain clinic and then physicians, it's not limited to physicians, but allied healthcare professionals in the community”* (Earth, AHP). Here, the participants mentioned the technological advantages of an ECHO (52)-like knowledge translation model already adopted across jurisdictions.

#### CAPHC Recommendation-6

The agreement on the involvement of PCPs was unanimous and participants felt that the benefits are mutual. Thus, AHP desired ongoing assistance from the PCPs, and clinicians pictured PCPs as a longitudinal “*safety net*” for AYAs in transition. Patient participants recommended the involvement of PCPs more as patients' allies and supporters during their adaptation period. PCPs desired that AYAs were “*…working closely… having regular contact with [their] primary care physician, that's something that often doesn't happen in chronic pain, people show up when they have an exacerbation, whereas this should be something that's managed on a regular basis with … a global treatment plan as opposed to a reactive type of plan”* (Dr. MDZERO).

#### CAPHC Recommendation-13

A pivot nurse position was another idea supported by all participants and discussed extensively in all stages. This role was seen as quite extensive and would include monitoring patient's attachment to primary care services and appointment attendance, ensuring the smoothness of ongoing communication between patients and involved healthcare professionals, informing and educating the patient, and be the first line of contact, “*like a resource person that people can call at all times…”* (Montreal, clinician). Patients hoped that the pivot nurse could absorb some of the physician's responsibilities, “*tell[-ing] you “You know what? Come into the hospital, I'm going to squeeze you in right away”…the root of the problem, is the fact that there is no person in the middle… and [there] has to be someone else in the middle, not the patient.”* (June, patient). The pivot nurse was seen as a patient advocate to “*communicate between primary care providers and… pain specialists”* (June, patient) and liaise with other professionals, “*be the go-between”* team member. (Jupiter, AHP). This would be “*…paramount because … unless you have that advocate or unless you can really advocate for yourself, not everyone has that makeup to really put themselves out there…”* (February).

Discussing professional collaborations, participants realized there might be some fundamental differences in how primary and specialty adult services are set up, which might be confusing for the AYAs, affecting their expectations from the services and different care providers. As one of the AHP group participants put it, “…*it speaks perhaps with the philosophy of family physicians vs. other physicians, where they're kind of cats vs. dogs. Where I think it's hard to herd cats. It's not a hierarchical structure as it would be in a hospital setting…So that you have to be careful with it*.” (Titan).

### Healthcare System Level

#### CAPHC Recommendation-14–15

All stakeholders agreed that there is a need for a consistent approach to transitional pain care pan-provincially. The tertiary care providers qualified most referral information as incomplete with “*the details there are grosso modo, they are just sort of ticked off”* (Rio, clinician). PCPs, on the other hand, described their confusion about pain services saying that “*there should be a site or something that gives us the adequate [information on the services]- because it doesn't go through the CRDS [central online depository], so you have to know where you're sending them.”* (Dr. Lily). One of the AYAs interviewed in the first phase exclaimed “*My family doctor? No, she had no idea how to help me and where to send me*.” Another patient recommended implementing “*a standardized transition system…Maybe a task force of different… doctors … who lay out their… 10 steps that need to happen when a patient aged 17 goes through for the next 12 months until they are kicked out [of pediatric services] and they start at the [adult] hospital.”* (February).

#### CAPHC Recommendation-17

Training for healthcare workers was another extensively discussed variable. For example, a patient supporter described her attempt to intervene “*[as she] saw a medical practitioner talking to July [AYA patient], who thought he had a 50-year-old, or a 40-year-old in front of him… [and supporter] was shut down because of the fact that she's obviously over 14.”* (May). An experienced educator AHP gave examples on his work with family medicine trainees evaluating AYAs who are “*different from older adults, who require different interviewing and management techniques…”* (Titan). A patient echoed these sentiments by emphasizing that gaps in training might be also associated with the perceived stigma: “*I just noticed that a lot of … doctors seem to be more kind of against like, “Oh, there's nothing wrong with you” or… they try but maybe they don't have the knowledge that they need…”* (March). The stigma of CP in a young patient was discussed by PCPs as something pervasive and omnipresent, one of the PCPs explained “*…[e]verybody will open the door for an old person with a cane or a walker, right, but a young person, everybody looks at them, and it's almost like they have leprosy. It's like there's something wrong with them, what did they do, you know”* (Dr. Lily). Another PCPs stated, “*I've been told that chronic pain is just as common in children and young people as it is in adults. I was very surprised by that information…”* (Dr. Diego).

A more detailed description of quotes and CAPHC-matched recommendations is found here (35).

## Discussion

We conducted a three-stage qualitative study on transitional pain services for AYAs in the McGill Healthcare Network and involved a diverse group of informants. The stakeholders discussed a complex interplay among the inter-disciplinary care provision, longitudinal primary care support, stigma, and other psycho-social determinants. The deliberative stakeholder consultation groups reviewed the findings of the first two steps, the transitional CAPHC guideline (R-1–3, 4–6, 9, 10, 13–15, 17) and formulated a set of 15 actionable recommendations (Table 1). The HCTRC theoretical framework was applied to formulate the vision for the transitional pain care model (Table 2).

**Table 1:** Summary of 15 actionable recommendations.

	**Individual level**	**Service level**	**Healthcare system level**
All three expert groups agreed	i) Building youth capacity, orientation to the adult pain services	ii) Development of a pivot nurse position iii) PCPs involvement iv) Multidirectional communication strategies	v) Standardized transition process across the province vi) Provincial e-platform to support transitions
Supported by two of *three* stakeholder groups	vii) Patient peer network	viii) Training in adolescent medicine for care providers ix) Strategies for accommodation of individual AYA's needs x) Overlap in care pre-transfer between specialists and PCPs / pediatric and adult providers xi) Age-friendly clinic design xii) Training in communication strategies for the clerical staff	
**Supported by one group**			
AHP		xiii) Increase in resources to provide specialist office hours for PCPs and psychology support for AYAs	
Clinicians		xiv) Direct contact with PCPs *via* e-consults, telephone helpline, and receiving preliminary information on incoming AYA patients	
Patients		xv) PCPs direct participation by attending AYAs' first adult appointment	

**Table 2:** TRAST transitional pain care model would involve all three levels.

**Individual level**
**∙ Programs and approaches to build AYAs' capacity post-transfer to adult pain services. For example, the development of a patient peer pain network to guide and support AYAs as they adapt to the adult services and acquire self-management and decision-making skills**. **∙ Increase in resources to provide support for AYAs in crisis. For example, additional psychology hours, a crisis line, and direct phone line with the pivot nurse**. **∙ AYAs-focused education therapeutic approaches. For example, group sessions on the coping skills and management of fibromyalgia**.
**Service level**
**∙ Development of a pivot nurse position/s to liaise with the community services, patients, and involved adult and pediatric specialists**. **∙ Longitudinal PCPs / primary care involvement to orchestrate the overall bio-psycho-social care plan, improve multi-directional information flow, and provide complementary care**. **∙ Multidirectional communication strategies between all stakeholders, including patients, primary, and specialty teams**. **∙ Ongoing training in adolescent and pain medicine for all healthcare professionals**. **∙ Strategies for accommodation of individual AYA's needs (drop-in hours, evening consultations, forms and certificate filling, etc.)** **∙ Continuous dynamic overlap in transitional care between pain specialists and PCPs, pediatric and adult providers**. **∙ Age-friendly pain clinic designs, allowing AYAs to meet their peers and mentors**. **∙ Training in communication strategies with AYAs for the clerical staff in healthcare institutions**. **∙ Increase in resources to provide pain specialist office hours for PCPs, direct contact with PCPs *via* e-consults, telephone helpline, and communication about preliminary information on incoming AYA patients**. **∙ Direct longitudinal PCPs' involvement in transitional pain care, including AYAs' first adult appointment. Provision of complementary (general medical) cares as an art of the transitional care for AYAs**.
**Healthcare System level**
**∙ There is an urgent need for a standardized transition and referral process across the province to clearly identify patients' trajectories and follow with the focused distribution of resources**. **∙ A common e-platform to support all transitions, facilitate information sharing, care coordination, and communication between all stakeholders**. **∙ Universal longitudinal post-transfer pain care for patients 16–25 years of age living with a CP diagnosis**. **∙ Inter-/multi-disciplinary set up with an overlap between pediatric, adult, and primary care services, which might include visiting clinicians-consultants from the relevant specialties**. **∙ Located in a relatively resource-rich environment that is connected to the local community, such as out-patient community hospital services or local community service centers (center local de services Communautaire's)**. **∙ Using standardized validated clinical tools and patient-focused material**. **∙ Functioning based on a defined set of relevant clinical and patient-focused outcomes, employing a written transition policy emphasizing a shared pre- and post-transfer responsibility for care**. **∙ “Soft” developmentally - appropriate transition deadline to exclude care fragmentation**. **∙ (Psychiatric, psycho-social, and addiction care mandate)?**

The main findings evolved around AYAs' pain experiences at three different levels, *individual* patient, *service*, and healthcare *system* levels. Interestingly, the focus of most discussions was on strategies with a direct impact on the AYA function within the *system*: building youth's capacity to navigate the system, orientation to adult pain services, development of a pivot nurse position to have a “go-to person,” PCPs' involvement in a role of an AYAs' ally when integrating into the new system, multidirectional communication strategies, standardization and predictability of the transition processes across the province, and implementation of the e-platform. This focus on the *system's* shortcomings likely stems from the AYAs' need for a more unambiguous and predictable environment that would limit “shifting sands” and allow for better function and coping.

Most but not all participants chose the development of a patient peer network, training in adolescent medicine for care providers, strategies on accommodations of individual AYA's needs, overlap in care, pre-transfer between specialists and PCPs/pediatric and adult providers, age-friendly clinic designs, and training in communication strategies for the clerical staff as important local initiatives that would strengthen transitional care. Resource-heavy recommendations (PCPs direct participation by attending AYAs' first adult appointment, increase in resources to provide specialist office hours for PCPs and psychology support for AYAs, direct contact with PCPs *via* e-consults, telephone helpline, receiving preliminary information on incoming AYA patients) were supported only by one enthusiastic group (patients, AHP and clinicians, respectively).

Participants, especially PCPs and AYAs, spent significant time discussing CP in young patients as being invisible, poorly understood by society, and not accommodated by the healthcare and educational systems. It was felt that this added layer of stress prevents AYAs from reaching their full potential resulting in poor psycho-social outcomes. The lack of resources for AYAs stems from the poor recognition of this vulnerable population, leading to the social insensitivity to pain in AYAs and its artificially diminished importance to society (7, 22). AYAs felt not believed and frequently dismissed as being unimportant, underscoring the need for a large scale significant educational efforts beyond healthcare providers that would reach different segments of society. With the involvement of decision-makers, focused funding for the development of pan-provincial transitional programs, e-platforms, and local resources, such as pivot nurses, will follow. The leitmotif of invisible pain in young patients was present in all discussions, thus stressing the urgent need to clearly define and recognize this population, in the same way as, for example, vulnerable elderly or children.

Suicide is already the second leading cause of mortality among Canadian youth (53) pre-pandemic. Although important as the individual patient-based outcomes, substance use and mental health were discussed only during the first two stages, but not during the project's final stage and thus, were not included in the final set of recommendations. It is possible that the discussion of some individual-level variables was regarded as rather secondary to a more global issue of the recognition and validation of CP experiences lived by AYAs in today's system. As the vision for the transitional framework starts taking shape, a more detailed and nuanced discussion of the individual factors will get more traction with the PCPs and AYAs presently struggling with the system imperfections, fragmentation, and severe lack of resources.

The need for accommodations in educational environments, pressure to obtain formal documentation, struggles to accomplish the same work as their peers, and desire to have additional opportunities for pain-related visits (drop off hours, telephone calls with a pivot nurse, on-line communication portal with team) underscored today fast-paced and highly competitive environment outside of the clinical setting that puts AYAs in a significant disadvantage socially but also medically. Patient supporters and PCPs admitted that AYAs are having difficulty planning, acting “in a reactive way” when the crisis has already arrived. Greig and Tellier described AYAs as those “…who are still in the process of acquiring autonomy, have marked similarities with adolescents and differ from older individuals who have attained full independence” (8). Furthermore, executive skills acquisition, such as planning, selective attention, and self-regulation, is fully attained around the mid-20s (54, 55) or even later in life, resulting in the need for more guidance and developmentally appropriate care (37) during this decade of transitions.

Sensitizing care providers to the existence of pain in AYAs and providing training in pain management were among the most discussed topics. To be able to recognize CP in AYAs, and properly assess and manage it, a curricula improvement for all healthcare professionals is urgently needed (56, 57). This might be easily achievable now with the explosion of web-based modules, applications, and online certificates. Yet, a common vision is required to structure educational efforts.

Multidisciplinary treatments are preferred but remain poorly accessible, and symptoms deteriorate while patients are waiting for clinic appointments (58, 59). Coupled with an imperfectly structured and understaffed system, the funding for sustainable models of transitional multidisciplinary services is critical for managing CP in AYAs. The rich insights can be gathered from the patient partners and their supporters, especially when they are provided with various advisory and governing opportunities within the same institutions, they get their treatments from. Development of the core outcomes in the psycho-socio-cognitive domains and decentralization of pain services with a shift away from the hospital-based services and closer to the AYAs' “medical home” (60–63) is an already existing vision (64).

## Data Availability Statement

The raw data supporting the conclusions of this article will be made available by the authors, without undue reservation.

## Ethics Statement

The studies involving human participants were reviewed and approved by St Mary's Hospital Ethics Review Board. McGill University. The patients/participants provided their written informed consent to participate in this study.

## Author Contributions

IK developed the original study idea, led the project, and wrote a draft of the manuscript. GB, MP, YS, LT, MC, and IV participated in all study steps, idea development, meetings, discussions, and conceptualization of the study findings, were involved in writing or proof-read the manuscript. All authors contributed to the article and approved the submitted version.

## Funding

The study received support from the Réseau de connaissances en services et soins de santé intégrés de première ligne du Québec (Réseau-1 Québec), grant # 29053. Louise and Alan Edwards Clinical Research on Pain fellowship support. *La Lettre* d'entente no 250 (recherche en médecine de famille) salary support.

## Conflict of Interest

The authors declare that the research was conducted in the absence of any commercial or financial relationships that could be construed as a potential conflict of interest.

## Publisher's Note

All claims expressed in this article are solely those of the authors and do not necessarily represent those of their affiliated organizations, or those of the publisher, the editors and the reviewers. Any product that may be evaluated in this article, or claim that may be made by its manufacturer, is not guaranteed or endorsed by the publisher.
